# Motor Adaptations to Pain during a Bilateral Plantarflexion Task: Does the Cost of Using the Non-Painful Limb Matter?

**DOI:** 10.1371/journal.pone.0154524

**Published:** 2016-04-26

**Authors:** François Hug, Paul W. Hodges, Timothy J. Carroll, Enrico De Martino, Justine Magnard, Kylie Tucker

**Affiliations:** 1 The University of Queensland, NHMRC Centre of Clinical Research Excellence in Spinal Pain, Injury and Health, School of Health and Rehabilitation Sciences, Brisbane, Australia; 2 University of Nantes, Laboratory EA 4334 “Movement, Interactions, Performance”, Nantes, France; 3 The University of Queensland, Centre for Sensorimotor Performance, School of Human Movement and Nutrition Sciences, Brisbane, Australia; 4 Sports Medicine Specialization School, Medicine, Surgery and Neurosciences Department, University of Siena, Siena, Italy; 5 The University of Queensland, School of Biomedical Sciences, Brisbane, Australia; University of Rome Foro Italico, ITALY

## Abstract

During a force-matched bilateral task, when pain is induced in one limb, a shift of load to the non-painful leg is classically observed. This study aimed to test the hypothesis that this adaptation to pain depends on the mechanical efficiency of the non-painful leg. We studied a bilateral plantarflexion task that allowed flexibility in the relative force produced with each leg, but constrained the sum of forces from both legs to match a target. We manipulated the mechanical efficiency of the non-painful leg by imposing scaling factors: 1, 0.75, or 0.25 to decrease mechanical efficiency (*Decreased efficiency experiment*: 18 participants); and 1, 1.33 or 4 to increase mechanical efficiency (*Increased efficiency experiment*: 17 participants). Participants performed multiple sets of three submaximal bilateral isometric plantarflexions with each scaling factor during two conditions (Baseline and Pain). Pain was induced by injection of hypertonic saline into the soleus. Force was equally distributed between legs during the Baseline contractions (laterality index was close to 1; *Decreased efficiency experiment*: 1.16±0.33; *Increased efficiency experiment*: 1.11±0.32), with no significant effect of Scaling factor. The laterality index was affected by Pain such that the painful leg contributed less than the non-painful leg to the total force (*Decreased efficiency experiment*: 0.90±0.41, *P*<0.001; *Increased efficiency experiment*: 0.75±0.32, *P*<0.001), regardless of the efficiency (scaling factor) of the non-painful leg. When compared to the force produced during Baseline of the corresponding scaling condition, a decrease in force produced by the painful leg was observed for all conditions, except for scaling 0.25. This decrease in force was correlated with a decrease in drive to the soleus muscle. These data highlight that regardless of the overall mechanical cost, the nervous system appears to prefer to alter force sharing between limbs such that force produced by the painful leg is reduced relative to the non-painful leg.

## Introduction

As a consequence of motor redundancy, the goal of a motor task can theoretically be achieved by a variety of motor strategies [1]. However, a limited set of solutions is typically used. For example, although multiple solutions are available to share load between the limbs during a bilateral task involving the homologous muscle(s), the preferred strategy is to share the force equally [e.g. bilateral plantarflexion [2,3] and bimanual tasks [4,5]].

Although a stereotypical motor strategy that shares the load between limbs is commonly used, the strategy can be modified by altering factors such as the efficiency of a component of the task. Hu and Newell [4] manipulated effector asymmetry during a bimanual force-matching task by imposing different scaling coefficients on force produced by the index finger of each hand. The unequal scaling coefficients altered the “strength” or the relative mechanical efficiency of the index fingers [increased efficiency of one side (e.g. force scaled by ×1.8) and decreased efficiency of the other (e.g. force scaled by ×0.2)]. Participants altered the force sharing strategy between index fingers such that the finger with the greater mechanical efficiency produced more actual force. This adaptation was interpreted to produce a more efficient motor strategy, i.e. more drive to the muscle with the larger force-generating capacity resulting in less total force actually exerted [4]. In these studies, participants were aware of the manipulation of limb efficiency and the imposed coefficients [4–6]. It is unclear whether participants would have adapted in the same manner in the absence of conscious awareness, i.e. with only an internal representation of the effort based on sensorimotor feedback and the efference copy of the motor output.

Changes in force sharing strategy are also provoked by nociceptive stimulation. When pain is induced by injection of hypertonic saline into one leg, the force produced by the painful leg is less than before pain during bilateral isometric plantarflexion [2] and squatting [7]. During these force-matching tasks with pain, the reduced force exerted by the painful leg is compensated by increased force produced by the non-painful leg [2,7]. This shift of load to the non-painful leg is hypothesised to be a purposeful strategy to reduce load within the painful region to protect from further pain and/or injury [8,9].

As the preferred strategy for a motor task is likely to depend on an interaction between competing trade-offs [10], the motor adaptations to pain might be affected by task constraints [7]. For example, modifying the “cost” of compensation with the non-painful leg might affect the amount to which the load is reduced within the painful region. This case would require tradeoff between the benefit (e.g. unloading, protection) and cost of adaptation in terms of effort or neural control (e.g. uncoupling the drive to the two limbs). Therefore, adaptation in force sharing between legs might be lessened if the mechanical efficiency of the non-painful leg were reduced such that unloading the painful leg incurs additional effort and control cost. Addressing this question is crucial to understand why reduced load within the painful region is not consistently observed [11–13].

Here we studied a bilateral task that allowed flexibility in the relative force produced with each leg, but constrained the sum of forces from both legs to match a target. We manipulated the mechanical efficiency of one leg by imposing different scaling factors on the force produced. We multiplied the force produced by one leg by 0.25 and 0.75 to produce a decrease in mechanical efficiency (*Decreased efficiency experiment)*, or by 1.33 and 4 to increase mechanical efficiency (*Increased efficiency experiment*). The primary aim was to test the hypothesis that the adaptation to pain (i.e. modified force sharing between legs) depends on the mechanical efficiency of the non-painful leg. More precisely, we tested whether increasing the cost of using the non-painful leg would reduce the amount of adaptation, and decreasing the cost of using the non-painful leg would increase the amount of adaptation. In order to test this hypothesis, we first determined whether force sharing between legs during a pain-free Baseline condition adapts to account for change in mechanical efficiency of one leg, in the absence of conscious awareness of this change. Myoelectric activity of agonist and antagonist muscles was recorded to determine whether the decrease in force produced by the painful limb was related to a decrease in drive to the painful muscle. In this case it would support the prediction of most pain theories that decrease in load within the painful region is a purposeful adaptation [8,9]. Alternatively, any decrease in plantarflexion force could be associated with an increase in co-activation of agonist and antagonist muscles. In this case, there would be no evidence to support the interpretation that adaptation to pain aims to decrease load in the painful tissue.

## Materials and Methods

### Participants

A total of 35 healthy volunteers participated in this study. Of these, 18 participated in the *Decreased efficiency experiment* (25±7 years, 69±10 kg, 173±8 cm, 6 females) and 17 participated in the *Increased efficiency experiment* (24±5 years, 62±9 kg, height: 169±8 cm; 8 females). The two experiments were performed on different participants to ensure that no interference was possible between experiments. Participants provided informed written consent. The local ethics committee (The University of Queensland, approval n° 2004000654) approved the experiment and all procedures adhered to the Declaration of Helsinki.

### Experimental set up

The experimental setup has been described in detail elsewhere [2] and was identical for the two experiments. Briefly, participants sat on a chair with the feet on separate force plates. The hip, knee and ankle were positioned at ~90° from full extension to limit contribution of gastrocnemii muscles to plantarflexion [14]. A horizontal bar that pressed against the distal thighs resisted movement of the legs during the isometric plantarflexion. To minimize movement of the body and changes in posture between contractions, the hips were fixed with a strap attached to the chair and the location of the feet was marked on the force plates.

### Force data

Separate force plates (Model 9260AA6, Kistler, Switzerland) measured the plantarflexion force produced by each leg. Data were sampled at 1 kHz (Power1401 Data Acquisition System, Cambridge Electronic Design, UK) and low-pass filtered (5 Hz, 4^th^ order Butterworth filter) off-line. Total plantarflexion force (Fz_tot_) was calculated as the sum of the left and the right plantarflexion force (Fz_L_ and Fz_R_, respectively) produced by the participants with no scaling factor applied. Effective plantarflexion force (Fz_eff_) was calculated as the sum of the left and right plantarflexion force after a scaling factor was applied (see below). For each condition, the target force required and feedback of Fz_eff_ was provided on a standard 21 inch computer screen ~ 1m in front of the participants at eye level. The temporal resolution was set at 10 s for full screen width, and the Y scale was optimized to the target force and remained the same for all scaling factor conditions.

### Electromyography data

Myoelectric activity was recorded bilaterally with surface electromyography (EMG) from the Soleus muscle (SOL) and its antagonist, the tibialis anterior muscle (TA). The SOL is the most mechanically efficient plantarflexor muscle when the knee is flexed at 90° [14]. For each muscle, a pair of self-adhesive Ag/AgCl electrodes (Blue sensor N, Ambu, Denmark) was attached to the skin with an inter-electrode distance of 20 mm (center-to-center). Before electrode application, the skin was cleaned with abrasive gel (Nuprep, D.O. Weaver & Co, USA) and alcohol. The ground electrode (half a Universal Electrosurgical Pad, 3M Health Care, USA) was placed over the right tibia. EMG data were pre-amplified 1,000 times, band-pass filtered on-line between 20–500 Hz (Neurolog, Digitimer, UK), and sampled at 1 kHz using a Power1401 Data Acquisition System with Spike2 software (Cambridge Electronic Design, UK).

### Experimental tasks

Before commencement of the experimental trial, two bilateral maximal isometric voluntary plantarflexions were performed for 3 s and separated by 90 s. If the total plantarflexion forces differed by more than 10%, a third contraction was performed. Maximum Fz_tot_ was defined as the maximum bilateral voluntary contraction force (MVC). The experimental task involved matching a target force set at 30% of MVC during ≈10–15 s isometric contractions, with 30 s rest between each repetition. Each participant received the following instruction: “*We need you to produce force to the target on the screen*. *The feedback you will receive is the sum of the force produced by both legs*. *We do not care about the balance between the legs*, *just the total force produced*.”

To decrease the mechanical efficiency of one leg, we scaled its force by 0.75 or 0.25 for the *Decreased efficiency experiment*. For example, when the force was scaled by 0.25, participants needed to develop 4 times the force to maintain the same contribution of this leg to Fz_eff_. For the *Increased efficiency experiment*, mechanical efficiency was increased by scaling the force by 1.33 or 4. For example, when the force was scaled by 4, participants needed 0.25 times the force to maintain the same contribution of this leg to Fz_eff_. Importantly, the participants were not informed of the scaling. Scaling was always applied to the leg that would not receive nociceptive stimuli (see *Experimental Pain*, below).

For both experiments, contractions with no change in scaling were considered as the control contractions. The no-pain condition (referred to as Baseline within the manuscript) started with three control contractions used to familiarize the participant with the task. Then, nine contractions (3 for each scaling factor) were performed in a randomized order. Following Baseline contractions, the contractions were repeated with pain. During the pain condition, two sets of three contractions (one for each scaling factor) were performed. The fewer number of sets during pain than baseline was motivated by the relatively short duration of pain (up to 10 minutes). The order of the three contractions within each set was randomized. As changes in motor control with pain do not necessarily resolve immediately after pain has ceased [15,16], the Baseline condition was systematically performed prior to the pain condition. At the end of the protocol the participants were asked: “*Did you feel that some contractions were of higher or lower intensity than others*?”

### Experimental Pain

**Fig 1:**
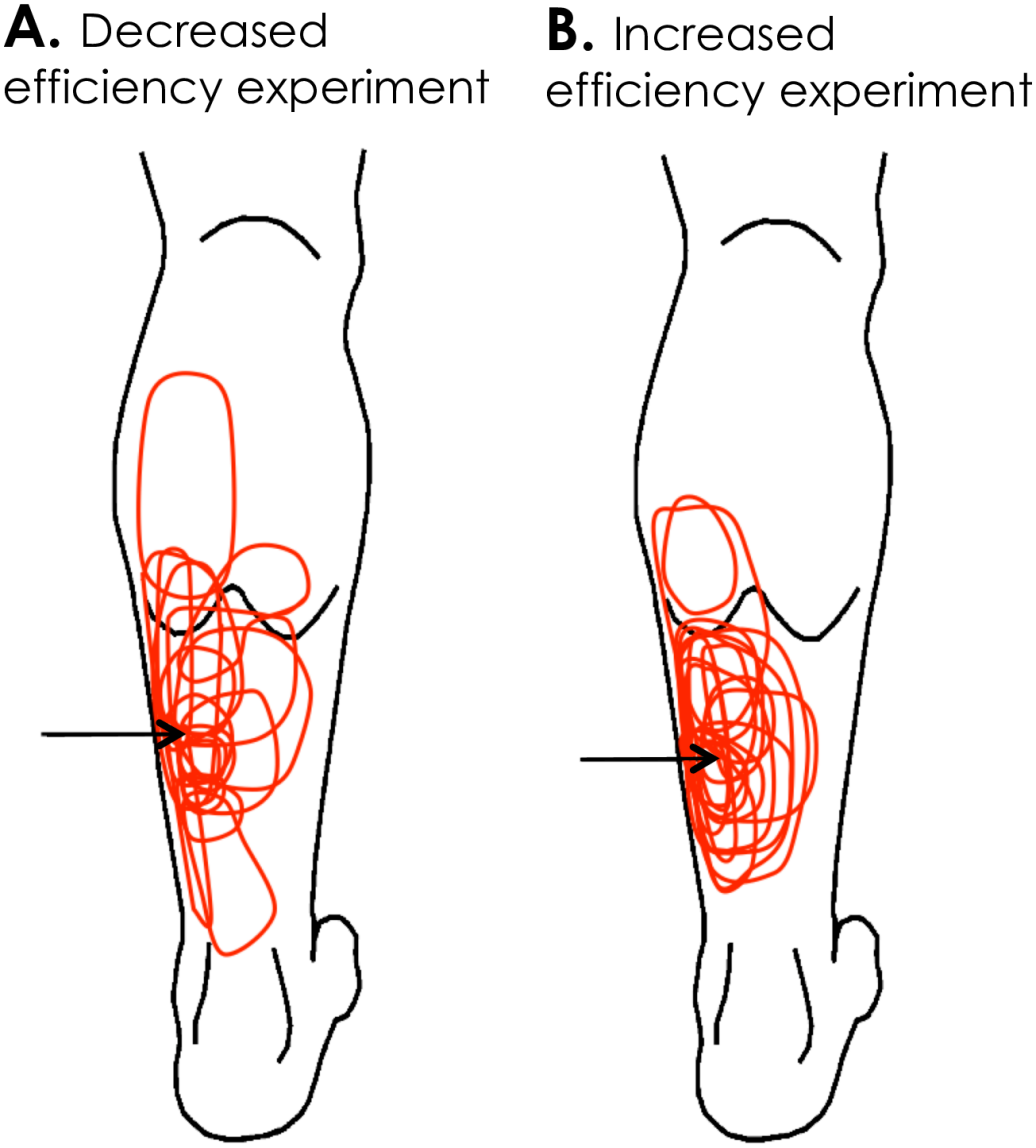
Area of perceived pain for each experiment. The injection site (arrow) and the area of reported pain for each participant (red) are shown. To enable comparison the pain location is overlaid on a left leg regardless of the actual side of injection.

Hypertonic saline (1 mL bolus 5% NaCl) was injected using a 25G × 19 mm hypodermic needle into the lateral soleus, approximately 1/3 of the distance from the ankle to the posterior knee crease. This location was confirmed by manual palpation during gentle plantarflexion efforts. The leg in which pain was induced was counterbalanced for each experiment. Participants rated pain intensity on an 11-point numerical rating scale (NRS), anchored with “no pain” at 0 and “worst imaginable pain” at 10. Contractions during the pain trial began after pain intensity was rated > 2/10. Immediately following each contraction, participants rated the pain intensity. After completion of the experiment, participants recorded the area of pain on a standardized diagram of the lower leg (Fig 1). Note that we have previously demonstrated that injection of non-painful saline (isotonic saline) does not alter the force sharing between limbs during a similar bilateral task [2]. Therefore, isotonic saline was not used as a control in our experiment.

### Data analysis

All data were processed using Matlab (The Mathworks, Nathick, USA). From each force-matched contraction, a 5-s window of data at the middle of the force plateau was used for analysis [7,11]. The Baseline force (i.e. weight of the legs and force applied though placement of the horizontal bar on the thigh) measured prior to each contraction was subtracted from subsequent contraction force data. To determine the force sharing between legs, we calculated the ratio of force, called “laterality index”: Fz _non scaled leg_/Fz _scaled leg_ [17]. In this way, a ratio of 1 indicates an even distribution of actual force between legs. If the contribution of the non-scaled leg was lower than the scaled leg, the ratio would be <1. The average amplitude of Fz_L_, Fz_R,_ Fz_tot_ and Fz_eff_ during each contraction was calculated. To determine how accurately the participants performed the force-matching task, we calculated the absolute error between the target force and Fz_eff_. The mean laterality index and absolute error were calculated over the 5-s window of each contraction. We further explored the possibility of changes in laterality index or absolute error over time within contractions, by comparing the start (average of the first 250 ms) and the end (average of the last 250 ms) of each 5-s window.

EMG amplitudes of SOL and TA were quantified as Root Mean Square (RMS) calculated over the same 5-s period as that used for the mechanical data. SOL EMG data were normalized to the peak EMG RMS measured during MVC contraction over a 500-ms window. The EMG laterality index was calculated for the SOL as RMS EMG _non scaled leg_/RMS EMG _scaled leg_ and used to assess the balance of neural drive between legs. As no maximal dorsiflexion was performed, TA EMG amplitude was not normalized to MVC and compared between conditions using un-normalized data (for the statistical analysis) or normalized data as percentage change from Baseline.

### Statistical analysis

Statistical analyses were performed using Statistica (Statsoft, USA). Distributions consistently passed the Shapiro-Wilk normality test and thus all data are reported as mean±SD. Because different subjects participated in the *Increased and Decreased efficiency experiments*, statistical analyses were performed for each experiment separately.

The force produced during the bilateral MVCs was first compared between legs using a paired *t*-test to verify that the force-generating capacity did not differ between legs. Then, for each scaling factor, within-session repeatability of the force sharing strategy was assessed between the 3 contractions performed during Baseline. To this end, both the Intraclass Correlation Coefficient (ICC) and the Standard Error of Measurement (SEM) of the force laterality index were calculated. Then, data were averaged across the contractions (i.e. three and two contractions for the Baseline and Pain conditions, respectively). To determine whether the mean absolute error between target force and Fz_eff_ was altered by pain and/or scaling factor, a repeated measure ANOVA was performed with Conditions (Baseline and Pain) and Scaling factors (control, 0.75 and 0.25 for the *Decreased efficiency experiment; and* control, 1.33 and 4 for the *Increased efficiency experiment*) as within subject variables. To determine whether this was modified throughout the 5-s contractions, another repeated measure ANOVA was performed with Time (start and end), Conditions and Scaling factors as within subject factors.

Pain intensity was compared using a repeated measure ANOVA with Scaling factor as a within subject variable. To determine whether the force sharing between legs during Baseline (no pain) adapted to account for the change in mechanical efficiency of one leg, we compared laterality index measured during Baseline using a repeated measure ANOVA with Scaling factor as a within subject variable. To satisfy the primary aim, the force laterality index, EMG laterality index, plantarflexion force of each leg (separately) and Fz_Tot_ were compared using separate repeated measures ANOVA, with Conditions and Scaling factors as within subject variables. To determine whether the laterality index of force changed throughout the 5-s period a repeated measure ANOVA was performed with Time (start and end), Conditions and Scaling factors as within subject variables. When required, post hoc analyses were performed using the Bonferroni test. Note that the pairwise comparisons of plantarflexion force were only considered within the same scaling condition, i.e. the force produced during pain for a given scaling factor was compared to the force produced during Baseline for the same scaling factor. P-values below 0.05 were considered significant (adjusted *P* values provided by the statistical software are provided in the *Results*). Cohen’s d values were calculated for the main outcomes (standard deviation of Baseline as the standardizer) as measures of effect size with 0.2, 0.5 and 0.8 as small, moderate and large effect, respectively [18].

Finally, to determine whether changes in plantarflexion force produced by the painful leg were associated with changes of SOL or TA activation, a correlation analysis was performed between change in plantarflexion force from Baseline and change in EMG signal amplitude from Baseline for each scaling factor condition.

## Results

Regardless the experimental condition (Baseline—when no pain was present, or Pain—immediately following the induction of pain), the leg in which pain was induced and to which no scaling was applied is referred to as the “painful leg” and the leg for which the force was scaled but to which no pain was induced is referred to as the “non-painful leg”.

### Maximal voluntary contractions

The contribution to bilateral MVC did not differ between legs (*Decreased efficiency experiment*: 50.2±4.4 vs. 49.8±4.4% of Fz_Tot_—*P* = 0.44; *Increased efficiency experiment*: 51.1±3.0 vs. 48.9±3.0% of Fz_Tot_—*P* = 0.14; for painful and non-painful legs, respectively). Regardless of which leg was selected for pain induction, the mean difference between the left and right legs measured during bilateral MVC was -2.8% and -2.5% of MVC for the *Decreased* and *Increased efficiency experiments*, respectively.

### Pain

For both experiments, pain was reported to originate within the soleus close to the site of hypertonic saline injection, except for one participant in the *Decreased efficiency experiment* who reported pain in the proximal calf (Fig 1). The averaged±*SD* pain intensity reported after each contraction was 4.8±1.5, 5.3±1.3 and 5.3±1.6 for control, scaling 0.75 and scaling 0.25, respectively (*Decreased efficiency experiment*) and 4.7±1.3, 4.8±1.5 and 4.6±1.3 for control, scaling 1.33 and scaling 4, respectively (*Increased efficiency experiment*). A main effect of Scaling factor was found for pain intensity in the *Decreased efficiency experiment* (F_2, 34_ = 5.39, *P* = 0.009). Post hoc testing resolved that pain intensity was higher when the efficiency of the non-painful leg was reduced compared to the control contractions (*P* = 0.037 [Cohen’s *d = 0*.*39*] and P = 0.015 [Cohen’s *d = 0*.*34*] for scaling 0.75 and 0.25, respectively). No effect of Scaling factor was found for *Increased efficiency experiment* (F_2, 32_ = 0.51, *P* = 0.78).

### Force performance

Regardless the experiment, the Scaling factor, or the Condition, the mean absolute error was low (< 5.9% of the target value; average = 2.7±1.2% of the target value), indicating that the participants performed all tasks well. However, some differences were observed. For the *Decreased efficiency experiment*, the mean absolute error was higher during Pain (2.6±0.2% of the target value) than Baseline (2.0±0.2% of the target value; F_1, 17_ = 4.71, *P* = 0.044; Cohen’s *d* = 0.80). Although significant, the increase in the absolute error was small (0.6% of the target value). There was neither a significant main effect of Scaling factor (F_2, 34_ = 0.95, *P* = 0.40) nor an interaction of Scaling factor × Condition (F_2, 34_ = 0.71, *P* = 0.50) for the mean absolute error. For the *Increased efficiency experiment*, there was a significant main effect of Scaling factor (F_2, 32_ = 4.29, *P*<0.0001). Post hoc analysis revealed that the mean absolute error was higher during scaling 4 contractions (4.7±1.7% of the target value) than during both control (2.2±1.0% of the target value; *P*<0.0001; Cohen’s *d* = 1.8) and scaling 1.33 contractions (2.3±0.9% of the target value; *P*<0.0001; Cohen’s *d* = 1.6). There was no significant main effect of Condition (F_1, 16_ = 2.19, *P* = 0.16), nor an interaction between Scaling factor and Condition (F_2, 32_ = 3.00, *P* = 0.06) for the mean absolute error in the *Increased efficiency experiment*

The absolute error did not change between the start and the end of the 5-s window in either experiment. Although there was a significant Time × Scaling factor × Condition interaction for the *Decreased* (F_2, 34_ = 6.41, *P* = 0.004) and the *Increased efficiency experiments* (F_2, 30_ = 4.63, *P* = 0.017), post hoc analysis did not show any significant change between Times (all P values > 0.3). The force laterality index did not change between the start and the end of the 5-s window during either experiment. Again, the significant Time × Condition interaction for the *Decreased* (F_1, 17_ = 7.84, *P* = 0.012) and *Increased efficiency experiments* (F_1, 16_ = 8.24, *P* = 0.012) was not associated with any significant changes between Times upon post hoc analysis (all P values > 0.4).

### Changes in force sharing in the absence of pain

During the control contractions performed during Baseline, the laterality index was close to 1 (1.16±0.33 and 1.11±0.32 for the *Decreased* and *Increased efficiency experiments*, respectively). For both experiments, repeatability of the laterality index between the 3 contractions performed within each scaling factor during the Baseline was fair to excellent (for both experiments: ICC ranged from 0.55 to 0.88; SEM ranged from 0.14 to 0.34).

**Fig 2:**
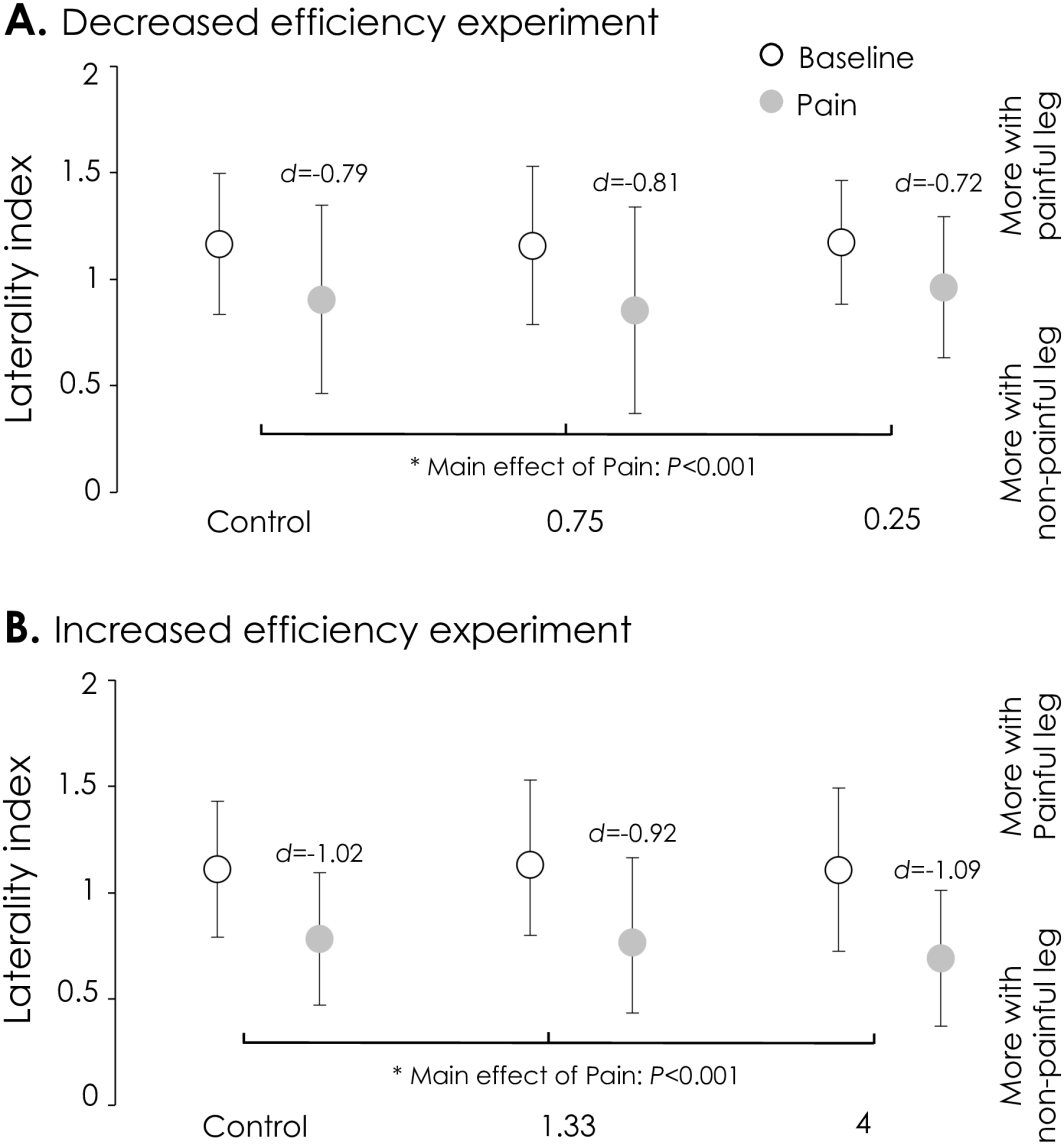
Laterality index. During Baseline, when the mechanical efficiency of the non-painful leg was manipulated (*Decreased efficiency experiment*, **A**; or *Increased efficiency experiment*, **B**), laterality index (distribution of force between the legs) did not change. That is, participants did not alter the force sharing strategy between the legs (open circles). Compared to Baseline, the contribution of the painful leg to total force decreased during pain (grey circles) for both experiments, and for all scaling factors. This decreased the laterality index during pain. *—P<0.05 for main effect of pain. Cohen’s *d* values calculated between no pain and pain are depicted for each scaling condition.

There was no main effect of Scaling factor on the laterality index, either when scaling decreased the efficiency of one limb (*Decreased efficiency experiment*: F_2, 34_ = 0.17, *P* = 0.84; all Cohen’s *d* values <0.07), or when scaling increased the efficiency of one limb (*Increased efficiency experiment*: F_2, 32_ = 0.18, *P* = 0.84; all Cohen’s *d* values <0.07; Fig 2). This result indicates that participants did not change the force sharing strategy between the legs when the virtual force-generating capacity of one of the legs was modified. Consequently, Fz_Tot_ (actual total plantarflexion force produced by the sum of both legs) produced during the Baseline condition was altered (main effect of Scaling factor: F_2, 34_ = 216.36, *P*<0.0001 and F_2, 32_ = 160.73, *P*<0.0001 for experiment I and II, respectively). For the *Decreased efficiency experiment*, Fz_Tot_ was higher when Scaling at 0.75 (33±2% of MVC; *P*<0.0001) and 0.25 (45±5% of MVC; *P*<0.0001) than control (29±2 of MVC) and higher for scaling 0.25 than 0.75 (*P*<0.0001). For the *Increased efficiency experiment*, Fz_Tot_ was lower for both scaling 1.33 (26±3% of MVC; *P*<0.0001) and 4 (13±2% of MVC; *P*<0.0001) than control (30±3% of MVC). Fz_Tot_ was also lower for scaling 4 than scaling 1.33 (*P*<0.0001).

According to their responses to questioning after the experiment, 11/18 and 14/17 participants consciously perceived a change in the total load of the task during the *Decreased* and *Increased efficiency experiments*, respectively.

### Changes in force sharing during pain

During both experiments, the laterality index was affected by pain (main effect Pain: *Decreased efficiency experiment*–F_1, 17_ = 16.20, *P*<0.001; *Increased efficiency experiment*—F_1, 16_ = 20.50, P<0.001). As there was no interaction between Pain × Scaling factor (*Decreased efficiency experiment*–F_2, 34_ = 1.31, *P* = 0.29; *Increased efficiency experiment*—F_2, 32_ = 1.23, *P* = 0.31) this indicates that the change in laterality index was similar, despite the manipulation of the efficiency of the non-painful leg (Fig 2). When averaged across the 3 scaling factors, the laterality index decreased from 1.16±0.32 to 0.90±0.41 (*Decreased efficiency experiment*) and from 1.12±0.36 to 0.75±0.32 (*Increased efficiency experiment*), which indicates that the force sharing strategy was altered by pain but did not depend on the mechanical efficiency of the non-painful leg.

Because of the difference in the mechanical efficiency between limbs, a similar change in the laterality index is not necessarily associated with a similar decrease in force (i.e. unloading of the painful tissue) across all scaling conditions. The change in laterality index indicates that the painful limb was less loaded than the non-painful limb, but this may not have led to a decreased load compared to Baseline (no pain) with the same scaling factor. To determine whether the painful limb was unloaded relative to Baseline we need to consider the plantarflexion force produced by the limbs independently.

### Changes in plantarflexion force during pain

#### Decreased efficiency experiment

**Fig 3:**
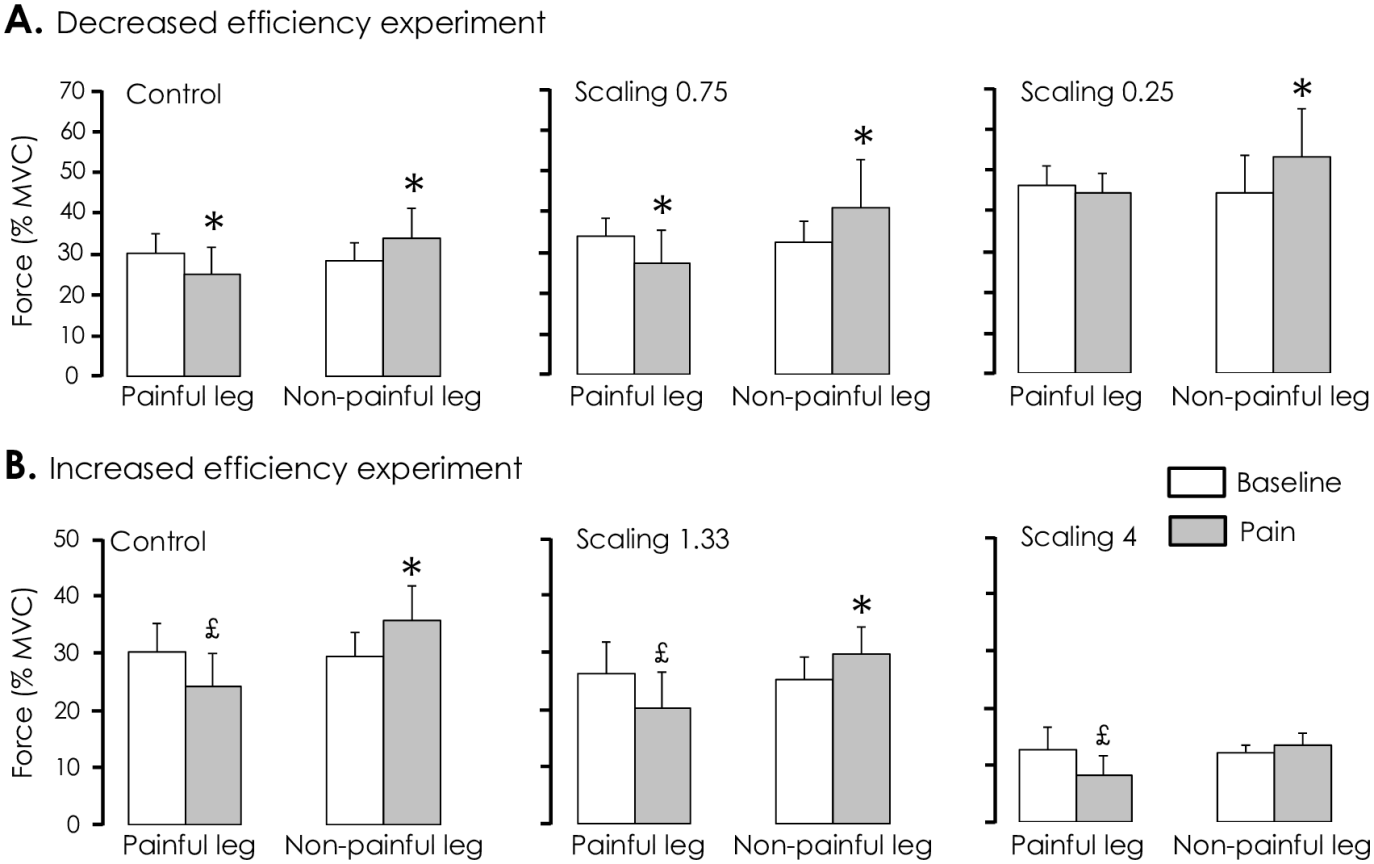
Actual plantarflexion force (N). Actual plantar flexion force generated by each leg is depicted for both experiments and each scaling factor. The force produced by each leg was compared between Baseline (white) and Pain (grey) within each scaling factor. *—P<0.05 for comparison to Baseline; £—P<0.05 for main effect of Pain for the painful leg; this indicates that the force produced by the painful leg decreased regardless of the scaling factor.

When the mechanical efficiency of the non-painful leg was decreased, the average plantarflexion force produced by the painful leg was affected by a significant interaction between Condition × Scaling factor (F_2, 34_ = 7.64, *P* = 0.002). For each scaling factor, the plantarflexion force produced during pain was compared to the force produced during Baseline of the corresponding scaling condition. As shown in Fig 3, the force produced by the painful leg was less during Pain than Baseline for both control (i.e. non-scaled) (*P*<0.0001, Cohen’s *d* = -0.34) and scaling 0.75 contractions (*P*<0.0001, Cohen’s *d* = -0.38). However, no reduction in plantarflexion force was observed in the painful leg when the mechanical efficiency of the non-painful leg was greatly decreased, i.e., for scaling 0.25 contractions (*P* = 1, Cohen’s *d* = -0.08).

There was a significant interaction between Condition × Scaling factor (F_2, 34_ = 3.41, *P* = 0.045) on the force produced by the non-painful leg. The force produced by the non-painful leg was higher during Pain than Baseline for all scaling factors (all *P* values<0.0006; Cohen’s *d* ranged from 0.69 to 1.12).

#### Increased efficiency experiment

When the mechanical efficiency of the non-painful leg was increased, there was a significant main effect of Condition (F_1, 16_ = 17.64, *P* = 0.0007) for the plantarflexion force produced by the painful leg. The force produced by the painful leg was less during Pain than Baseline regardless the mechanical efficiency of the non-painful leg (Fig 3). The effect size was moderate to large (Cohen’s *d* = -0.68, -0.65 and -0.94 for Control, scaling 1.33 and scaling 4, respectively). Although close to the threshold of significance, there was no significant interaction between Condition × Scaling (F_2, 32_ = 2.64, *P* = 0.084), indicating that there was similar adaptation to pain regardless of the degree of increased mechanical efficiency of the non-painful leg.

Considering the force produced by the non-painful leg, there was a significant interaction between Condition × Scaling factor (F_2, 32_ = 13.89, P<0.0001). The force produced by the non-painful leg was higher during Pain than Baseline for both control (P<0.0001, Cohen’s *d* = 0.72) and scaling 1.33 (P<0.0001, Cohen’s *d* = 0.72). No difference was found for scaling 4 (P = 1, Cohen’s *d* = 0.43).

### Relationship between muscle activation and plantarflexion force

**Fig 4:**
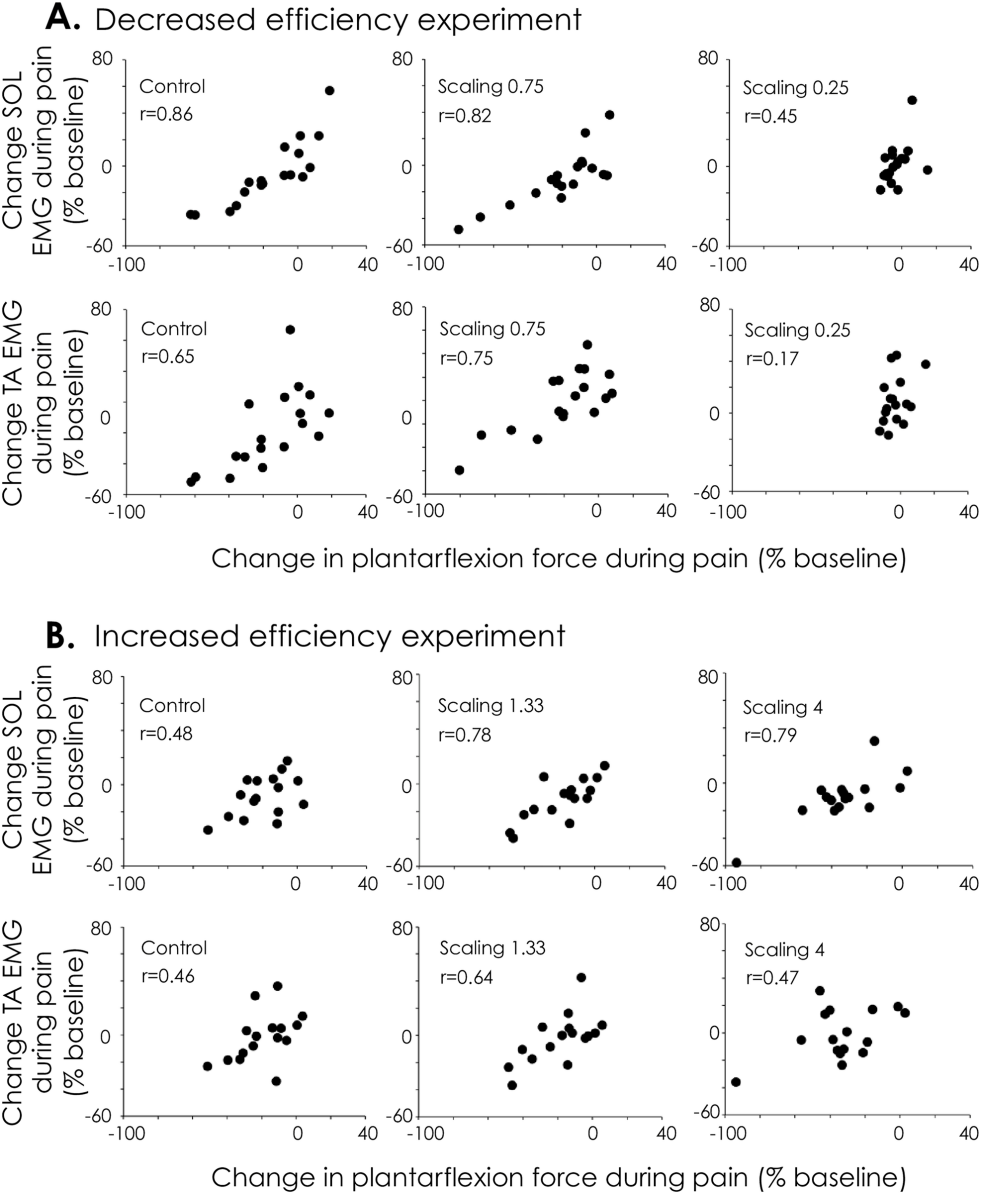
Correlation between changes in plantarflexion force produced by the painful leg and changes in EMG amplitude of both agonist (soleus: SOL) and antagonist (tibialis anterior: TA) muscles. For both experiments, a moderate to strong positive correlation was found between change in force produced by the painful limb and change in EMG amplitude of soleus and tibialis anterior. This indicates that the decrease in force observed during pain (with the exception of scaling 0.25) was explained by decreased drive to the main plantarflexor muscle and its antagonist.

For both experiments, a moderate to strong positive correlation was found between change in force produced by the painful limb and change in SOL EMG amplitude (r values ranged from 0.45 to 0.86; Fig 4). This indicates that the decrease in force observed during pain (with the exception of scaling 0.25) was explained, at least in part, by decreased drive to the main plantarflexor muscle. Similarly, a moderate to strong positive correlation was found between changes in force produced by the painful limb and changes in antagonist muscle activity (TA; r values ranged from 0.46 to 0.75; Fig 4) for all conditions/scaling factors, except scaling 0.25 (r = 0.17). This positive correlation indicates that antagonist activity decreased as force produced by the painful leg decreased.

Note that the EMG laterality index calculated from SOL exhibit the same changes with pain as those reported for the laterality index calculated from plantarflexion force. During both experiments, the EMG laterality index was affected by pain (main effect Pain: *Decreased efficiency experiment*–F_1, 17_ = 6.98, *P* = 0.017; *Increased efficiency experiment*–F_1, 15_ = 13.72, P = 0.002) and this change in EMG laterality index was similar, despite the degree of manipulation of the efficiency of the non-painful leg. When averaged across the 3 scaling factors, the laterality index decreased from 1.14±0.39 to 0.92±0.42 (*Decreased efficiency experiment*) and from 1.04±0.25 to 0.85±0.27 (*Increased efficiency experiment*) during pain.

## Discussion

We aimed to determine whether the motor adaptation to nociceptive input/pain is influenced by the mechanical efficiency of the non-painful leg. We did this by investigating potential changes in laterality index (i.e. force sharing) between legs as a measure of motor adaptation in situations where the mechanical cost of adaptation was increased or decreased. Our study has two major findings. First, during pain-free contractions, our data show that if participants are not informed of a manipulation of virtual mechanical efficiency of one limb, they maintain near equal force generation with both legs. Second, during nociceptive stimulation in the control condition, and regardless of the virtual change in mechanical efficiency of the non-painful leg, the sharing of force between legs was altered such that some plantarflexion force was transferred from the painful limb to the non-painful limb. The decrease in plantarflexion force produced by the painful leg was positively correlated with a decrease in drive to the soleus muscle. A decrease in force produced by the painful leg during pain was observed for all conditions, except when the efficiency of the non-painful limb was greatly decreased (scaling 0.25). Taken together, these data highlight that regardless of the overall energy cost, the nervous system appears to prefer to reduce the force produced by the painful leg relative to the non-painful leg. This supports the notion that protection of painful tissue is prioritized by the nervous system.

### Force sharing is not affected by an undeclared unilateral change in mechanical efficiency (in the absence of pain)

It has been proposed that the central nervous system controls force sharing to be optimal in the sense that it minimizes costs such as energy expenditure or force variability (for review see Prilutsky and Zatsiorsky [19]; but cf. de Rugy et al. [20] for an alternative perspective). Although the idea that the CNS seeks to optimise force sharing between limbs is supported by previous studies in which modifications to efficiency were known to the participants [4,5,21], there was no change in laterality index in the present study when the mechanical efficiency of one leg was increased or decreased without participants being explicitly informed of the change. There are several possible interpretations of this finding.

First, the controller may not necessarily optimise force sharing to minimize mechanical cost, and other “costs” may have been prioritised. For instance, from a neural control perspective there may have been additional cost associated with decoupling of the forces between legs. Although dissociation of the output between legs in the present paradigm was clearly possible, it was not observed in the absence of pain. Maintenance of similar outputs from both limbs in a bilateral task may be simpler to control than producing asymmetric outputs to each leg. Greater “control costs” associated with asymmetric drive could have several neural bases. For instance, bilateral efforts involve some degree of common drive to both limbs [22]. During bilateral tasks, particularly those of a postural nature, the soleus muscle of each limb receives some common drive which results in synchronised discharge of motor units [23]. In this way, some have suggested the majority of drive to both limbs in submaximal bilateral efforts may be controlled by a single cortical hemisphere [22], and separation of control between limbs would impose a control cost.

Second, the absence of adaptation in the present study could be explained by an inability of participants to detect a unilateral change in efficiency, in the absence of explicit information about this modification, rather than a choice to not compensate. In contrast to previous work [4,5,21], participants were not informed that mechanical efficiency could differ between limbs in the present study. Although most participants detected a change of the total force demand due to scaling, it is possible that they did not detect the difference in efficiency between limbs. As participants were only provided feedback of total force, identifying an efficiency asymmetry would have required complex integration of visual feedback of bilateral force with perceived effort of contraction in each limb. Therefore, the lack of adaptation might be explained by a failure to identify that the change in total demand of the task was related to changes in the mechanical efficiency of a single limb.

Third, the lack of adaptation in the present study might be explained by the limited exposure to the change in mechanical efficiency. However, this short testing period was sufficient to observe systematic adaptations in force sharing when challenged by pain.

Overall, even if our results cannot definitely address the question on how the nervous system adapts to change in mechanical efficiency of one leg (which was not our primary aim), we manipulated the required force (cost) of compensation to pain and therefore, the protocol was still valid to determine whether adaptations to pain depend on the cost of using the non-painful limb.

### Force sharing is modified during pain, regardless of the mechanical efficiency of the non-painful limb

We used an isometric bilateral task to study pain adaptation as it offers an obvious solution to alter force sharing and thus load within the painful limb. A systematic shift in force toward the non-painful limb during force-matched bilateral tasks has been shown [2,7]. In line with these results, a significant change in the laterality index leading to decreased (~16–20%) force produced by the painful leg was observed during the control contractions (when efficiency of the non-painful leg was unchanged) for both experiments. Notably, this reduction in the laterality index was observed for all conditions, regardless of mechanical efficiency. This means that regardless of the additional energy cost associated with bias of force towards the non-painful limb, the change in strategy was similar.

The motor adaptation to pain is thought to purposefully reduce load within the painful tissue [8,9]. This conjecture is supported by the present finding that decreased plantarflexion force of the painful leg was associated with decreased drive to the SOL muscle. However, we report considerable variability in the change in SOL and TA muscle activity between individuals (highlighted in Fig 4). This variability in motor adaptation is consistent with other pain literature (e.g. [24,25]). In addition, although the force produced by the painful leg was systematically reduced relative to the non-painful leg, the force produced by the painful leg was not always significantly less than the same condition (same modification to mechanical efficiency) without pain (during Baseline). That is, the painful limb was not unloaded relative to the force required to perform the task for scaling 0.25 in the *Decreased efficiency experiment*. This observation has several possible interpretations.

First, in the context of a bilateral effort that normally involves equal force production with both legs, the nervous system may perceive a reduction in force *relative* to the contralateral homologous muscle as beneficial for tissue loading. In view of the theory of pain proposed by Wall et el. [26], this act of having “taken action” to reduce the threat by generating less force than the contralateral muscle, despite a failure to reduce the load relative to a pain-free condition, may be perceived by the nervous system as beneficial.

Second, a failure to unload the painful leg when the non-painful leg was scaled by 0.25 might be associated with an unacceptably high “control” cost due to the requirement to dramatically decouple drive to the two legs in the bilateral task. For any given decrease in force produced by the painful leg (i.e. to unload the painful tissue) in this case, participants would have had to increase the force produced by the non-painful leg four-fold. Specifically, to maintain the task while accommodating a force reduction equivalent to the average pain adaption observed during the control condition (40 N), an extra 160 N would be required from the non-painful leg. In this case, the laterality index would have decreased to ~0.6, which is lower than the lowest values observed during the two experiments. This high “control” cost hypothesis is in-line with results of a previous study on bimanual force-matching task where task asymmetry was manipulated by imposing different scaling factors on force produced by the index fingers of each hand [6]. The changes in force sharing were relatively limited (the laterality index between sides ranged between 0.74 and 1.47) although the scaling coefficient ratios ranged twenty-fold. These authors also showed that, as the weighting coefficients became more uneven, force output ratios tended to plateau [5]. The potential for a “control” cost to increase as the laterality index moves away from 1 (i.e. the load sharing becomes more uneven) could also explain why the increased efficiency of the non-painful leg did not trigger further changes during the *Increased efficiency experiment*. In the *Increased efficiency experiment*, the shift of the force toward the more efficient non-painful leg was an efficient strategy both to unload the painful leg and to decrease the overall energetic demand of the task. However, the *optimal* strategy here would have been to drastically decrease force produced by the painful leg and produce the majority of force with the more efficient leg. Instead, participants maintained the same laterality index that was observed during control contractions with pain.

Third, the reduction in force produced by the painful limb may be related to an altered internal representation of the force produced during pain. When pain is induced in one arm during a bilateral force-matching task, the force produced by the painful limb is overestimated, leading participants to produce less force in the painful than the non-painful limb [27]. In this case, the consistent change in laterality index (i.e. force sharing) may be explained by an overestimation of force produced by the painful limb, and an attempt to maintain equal force between legs, rather than a *purposeful* adaptation to unload the painful tissue. Our observations of a consistent proportional change in laterality index, and a failure to consistently unload the painful limb with extreme changes in efficiency, supports this hypothesis and requires further investigation.

Note that pain intensity was higher when the mechanical efficiency of the non-painful leg was less than the control contractions. However, the size of this affect was small to moderate (<0.5/10; Cohen’s d <0.39). We are unaware of evidence that pain levels differ systematically depending on force produced by a painful muscle, particularly within the range of 30–45% of MVC (as was the case in the current *Decreased efficiency experiment*). Rather, we previously found no systematic differences in reported pain levels when contraction intensity ranged from 10 to 70% of MVC, and between contraction and rest [2]. With this in mind, we do not interpret the small differences in pain intensity reports to be of practical significance.

## Conclusion

These data provide evidence that sharing of force between legs is altered during unilateral pain, independent of the mechanical efficiency of the non-painful leg. Although the painful limb always produced less force than the non-painful limb in a given contraction with pain, the constant change in force sharing observed between limbs, under various experimental manipulations of relative limb efficiency, did not ensure a systematic unloading of the painful tissue relative to corresponding pain free trials. These results have implications for the evolving theory of pain adaptation. There may be a trade-off between a perceived benefit of unloading the painful part (e.g. decreased pain, protection from further injury) and additional costs related to potential motor adaptation options. In the current study, the additional cost presumably relates to the dissociation of drive between the legs during the bilateral task and/or the overall increase in force level. An alternative explanation, related to the change in internal representation of the force produced during pain, requires further investigation.

## Supporting Information

S1 Table Force data for the *decreased efficiency experiment*. Data are presented in Newtons. Note that regardless the experimental condition (Baseline—when no pain was present, or Pain—immediately following the induction of pain), the leg in which pain was induced and to which no scaling was applied is referred to as the “painful leg” and the leg for which the force was scaled but to which no pain was induced is referred to as the “non-painful leg”.
(XLSX)

S2 Table Force data for the *increased efficiency experiment*. Data are presented in Newtons. Note that regardless the experimental condition (Baseline—when no pain was present, or Pain—immediately following the induction of pain), the leg in which pain was induced and to which no scaling was applied is referred to as the “painful leg” and the leg for which the force was scaled but to which no pain was induced is referred to as the “non-painful leg”.
(XLSX)

S3 Table Laterality index data for the *decreased efficiency experiment*. The “laterality index was calculated as: Fz non scaled leg(painful leg)/Fz scaled leg(non painful leg). Note that for some conditions, some participants performed only one contraction with pain. It is because pain decreased <2/10 before the second repetition.
(XLSX)

S4 Table Laterality index data for the *increased efficiency experiment*. The “laterality index was calculated as: Fz non scaled leg(painful leg)/Fz scaled leg(non painful leg). Note that for some conditions, some participants performed only one contraction with pain. It is because pain decreased <2/10 before the second repetition.
(XLSX)
